# Quality and Safety of Ready-to-Eat Golden Thistle (*Scolymus hispanicus* L.): A New Product for Traditional Italian Dishes

**DOI:** 10.3390/plants12081622

**Published:** 2023-04-12

**Authors:** Lucrezia Sergio, Donato Di Venere, Maria Gonnella, Massimiliano D’Imperio, Federico Baruzzi, Loris Pinto, Francesca Boari, Vito Cantore, Vincenzo Candido

**Affiliations:** 1 Institute of Sciences of Food Production, National Research Council of Italy (CNR), Via G. Amendola 122/O, 70126 Bari, Italy; lucrezia.sergio@ispa.cnr.it (L.S.); maria.gonnella@ispa.cnr.it (M.G.); massimiliano.dimperio@ispa.cnr.it (M.D.); federico.baruzzi@ispa.cnr.it (F.B.); loris.pinto@ispa.cnr.it (L.P.); vito.cantore@ispa.cnr.it (V.C.); 2 Department of European and Mediterranean Cultures, University of Basilicata, Via Lanera, 10, 75100 Matera, Italy; vincenzo.candido@unibas.it

**Keywords:** antioxidants, cooking, edible herbs, nitrates, polyphenols, safety, sensory evaluation, sugars, wild species

## Abstract

Golden thistle (*Scolymus hispanicus* L.) is a wild edible plant belonging to *Asteraceae* family, with a great potential for food applications. The aim of this study was to identify the best cooking procedure able to provide a high-quality, ready-to-use product. For this purpose, leaf midribs (the most used edible part of the plant) were cooked by boiling, steaming, and ‘sous vide’, and the cooked products were compared for their phenolic content and composition, antioxidant activity, sugar and inorganic ion content, organoleptic characteristics, and microbial safety, this latter also during storage. In general, boiling caused a decrease in the value of these parameters, despite being the best product for taste and overall acceptability. On the contrary, steaming and ‘sous vide’ resulted in the best treatments to preserve antioxidant activity, total phenols, and chlorogenic acid. In particular, in ‘sous vide’ cooked samples, a significant increase in the value of these parameters and a remarkable decrease in nitrate content were found. Moreover, ‘sous vide’ resulted in the best treatment also regarding microbial safety during shelf life; actually, after 15 days of storage at 8 °C, *Enterobacteriaceae* and mesophilic aerobic bacteria were not detectable in ‘sous vide’ samples. These results contributed to increase the knowledge of a wild edible plant with high nutritional properties and promoting its consumption by obtaining a ready-to-use product with good organoleptic characteristics and endowed with a long period of shelf life.

## 1. Introduction

In the last decades, a strong desire to recover past culinary traditions, rediscover ancient flavors, expand the range of food preparations, and mostly consume healthy products has spread among the people [1,2]. Thus, the historical relationship between territory and traditional food transformation has been defined as “food heritagisation” [3]. Many recipes that meet these requirements are based on the use of wild edible plants. Unfortunately, the recognition of these herbs and methods of preparation requires very special skills, which are now the prerogative almost exclusively of elderly people and that rarely are taught to young people. In addition, these preparations often take a long time, which is not compatible with the rhythms of work and, more in general, with the modern lifestyle.

The use of edible wild plants in cooking is a common practice in Italy [4,5,6,7]. In the Puglia region (Southern Italy), there are numerous (about 2500) wild herbaceous species, 532 of which are consumed as food [8,9]. Depending on the plant, leaves (for 80% of species), tender stems (36%), and flowers (20%) are edible, although more than 90% of these parts are consumed after boiling [10].

Wild edible plants have been an important resource during times of food scarcity, but nowadays, they are consumed for the pleasure of harvesting and rediscovering ancient flavors, as well as for their nutritional value, as they are rich in vitamins and minerals. The therapeutic and nutritional properties of wild edible plants have been the subject of numerous studies [11,12,13,14] because they are generally characterized by high nutritional value and low energy content [15]. Moreover, the preservation of nutritional properties of these herbs is largely determined by the cooking process, which can affect antioxidant activity prevalently due to changes occurring in phenolic composition [14].

**Figure 1:**
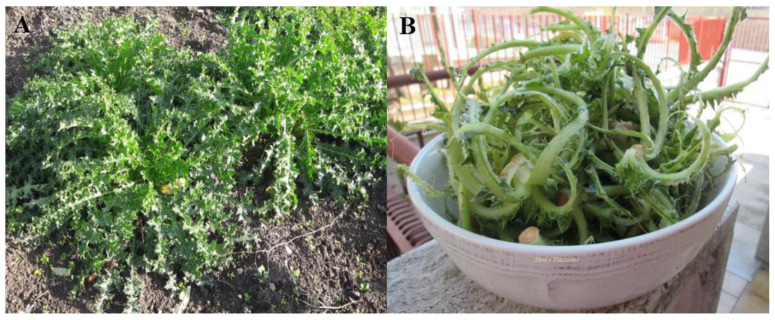
(**A**) Golden thistle in field; (**B**) the edible parts of the plant: midribs (central leaf veins). (image (**B**): Credits: https://dolcipassioni.net/wp-content/uploads/2021/04/mar283-2.jpg, accessed on 20 March 2023).

Golden thistle (*Scolymus hispanicus* L.) (Figure 1A) is a prickly perennial herbaceous plant of the *Asteraceae* family. It is native to Southern Europe (Portugal, Spain, France, Italy) and Western Asia (Iran); it is a mild climate plant growing in Mediterranean countries, including the Aegean and Marmara regions (Turkey) [16,17]. It can be found in uncultivated areas along roadsides. The edible parts of golden thistle are midribs (i.e., the central leaf veins, Figure 1B), petioles, and roots that, according to the traditional Mediterranean cuisine, are usually eaten boiled, mashed, and baked, while the young tender leaves and blanched leaf stalks are consumed as a fresh salad dressed with extra virgin olive oil, salt, and vinegar [18,19].

Even though it is employed as an ingredient in several Christmas and Easter dishes, it has recently taken a considerable interest for its anti-bacterial, anti-herpetic, anti-inflammatory, anti-spasmotic, anti-tumor effects on colon, kidney, and lung cancers [20,21,22], as well as anti-sweat and diuretic properties [23]. Moreover, it is considered a good source of calcium, potassium, and magnesium [24,25,26], and it was found to be rich in several nutritional and bioactive compounds. Indeed, some authors reported the presence in the golden thistle of a low total fat content, good levels of proteins and dietary fiber [25,27], carbohydrates [27], and organic acids (i.e., oxalic, malic, quinic, shikimic, citric) [22,27], together with remarkable content of flavonoids, phenolic acids, and tocopherols [22,23], and poly-unsaturated fatty acids (in particular, linoleic acid) [27].

Currently, in Italy, as well as in other Mediterranean areas [26], the golden thistle is not a cultivated plant; thus, local markets are supplied by collectors of wild-growing plants at the most suitable time for their consumption. Plants available as spontaneous in nature can originate from the re-growing of plants from the previous year or from seeds. In any case, the period and the number of harvests depend primarily on the rainfall regime occurring between August and October. In particular, in the case of plentiful rainfall between the end of August and September, it is possible to carry out a first harvest at the end of November and a second one later in spring. The increased demand from consumers has led to an increase in studies on the domestication of this species [26,27,28,29,30,31,32], as well as on the preparation of ready-to-use products and food additives [17].

To the best of our knowledge, the scientific literature does not report studies concerning golden thistle cooking procedures addressed to obtain a ready-to-eat product. The aim of this study was to fill this gap by providing experimental results focused on realizing a product with a long shelf life that can be used as it is, simply seasoned, or that can be used included in more complex traditional Mediterranean recipes (‘au gratin’, with lamb, with pasta). The availability of a new ready-to-eat wild plant also opens for new and more elaborate recipes and dishes.

For this purpose, boiling, steaming, and ‘sous vide’ cooking methods were compared, characterizing the ready-to-use cooked products for their phenolic content and composition, antioxidant activity, as well as inorganic ion and sugar content. Moreover, products were evaluated for their organoleptic characteristics and microbial safety, later also during storage.

## 2. Results and Discussion

### 2.1. Dry Matter of Raw and Cooked Golden Thistle

The dry matter (DM) of the raw edible portion of golden thistle was 5.7 ± 0.1 g 100 g^−1^ fresh matter (FM) and did not change statistically after steaming or ‘sous-vide’ cooking. In contrast, boiling caused a 17.5% reduction compared to the raw product (Table 1), probably due to solute loss by leaching and particle leakage from the tissue in hot water. This effect of boiling was also observed in chicory and asparagus with different relevance depending on the vegetable [33,34]. After cooking, a significant weight change occurred in all treatments, although to a different extent, through weight loss, certainly due to the loss of water and solutes during cooking (Table 1). This was more consistent in the steamed and ‘sous vide’ samples (−9.2%, on average) than in the boiled ones. In other studies, the boiling treatment resulted in minimal weight loss [33] or even positive weight change, denoting the occurrence of a slight imbibition process [34].

### 2.2. Inorganic Anion Content

The nitrate content halved, on average, after cooking treatments (−43.3% compared to 69.1 ± 2.3 mg g^−1^ DM of the raw product). Similarly, the content of Cl^-^ and H_2_PO_4_^-^ consistently decreased after boiling, confirming the hypothesis of loss of solutes as a consequence of cooking. The phosphate content showed the highest reduction in ‘sous-vide’ treatment; instead, the sulfate content did not change between raw and cooked products, remaining about 1.0 mg g^−1^ DM (Table 1). Expressed as fresh matter-based value, the nitrate content of golden thistle midribs appears quite high for a wild edible plant. In the raw product, it reached more than 3900 mg kg^−1^ FM. It was more than halved after boiling, but the decrease was lower after ‘sous-vide’ (−42.6%) and steaming (−37.7%) treatments. The nitrate content found by Disciglio et al. [24] in the edible portion of golden thistle was near 500–600 mg kg^−1^ FM. The comparison between the two values cannot be made, as Disciglio et al. [24] have used samples of wild golden thistle and therefore not fertilized, while our plant material came from an experimental trial of domestication in which golden thistle had been fertilized with 100 kg ha^−1^ N. Furthermore, it is difficult to compare the results of the two studies as the nitrate content in the plants is a parameter greatly influenced by climatic conditions (temperature and solar radiation) [35,36]. On the other hand, the cultivation of golden thistle under optimal nutritional conditions made its leaves rich in nitrate at the same level as our cultivation, causing an accumulation of nitrate in leaves up to 3750 mg kg^−1^ FM [26].

The golden thistle is not comparable as a vegetable product to any species among those included in the EU Regulation n. 1258/2011, which fixed the maximum levels of nitrate. According to the EU Regulation, the nitrate content in spinach cannot exceed the maximum level of 3500 mg kg^−1^ FM; however, in lettuce, up to 5000 mg kg^−1^ FM are allowed during winter for lettuce obtained in a greenhouse. In rocket, the limit is considerably higher (6000–7000 mg kg^−1^ FM) due to the difficulty of obtaining a lower accumulation of nitrate in rocket products. In the case of golden thistle, which belongs to the same botanical family as lettuce (the genotype is one of the main factors influencing the accumulation of nitrate in vegetables) and tends to accumulate nitrate at a high level, two further elements must be considered: on the one hand, the edible portion is the midrib, which represents the organ of the greatest accumulation of nitrate in the plant; on the other hand, the edible product can be consumed only after a cooking treatment, favoring a consistent loss of nitrate from the edible part. Since the average amount of nitrate found in the cooked product was less than 2200 mg kg^−1^ FM, it can be estimated that a 100 g portion of golden thistle provides a maximum nitrate intake of 220 mg, i.e., a value safe for a 60 kg adult, taking into account that the maximum value of 3.7 mg kg^−1^ body weight per day is the recommended Acceptable Daily Intake (ADI) for nitrate [37]. Finally, this aspect must be kept in mind when defining the cultivation technique of this species if one wishes to elevate it to a cultivated species.

### 2.3. Total Phenols and Antioxidant Activity

**Figure 2:**
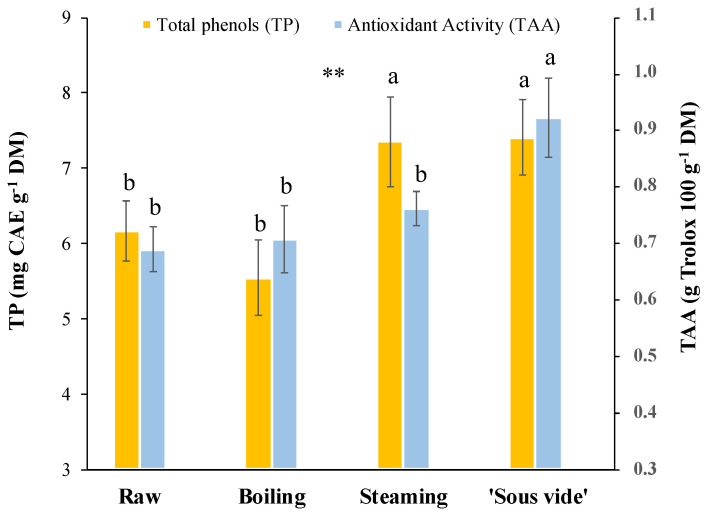
Total phenols (TP) content and total antioxidant activity (TAA) in golden thistle midribs raw and cooked by boiling, steaming, and ‘sous vide’. Vertical bars indicate the standard deviation. Different letters indicate statistically different values (α = 0.05) between treatments. Significance: **, significant at *p* ≤ 0.01.

The TP content and TAA, both in the raw as well as cooked samples, are reported in Figure 2. In the raw product, values of 6.2 ± 0.4 mg CAE g^−1^ DM and 0.7 ± 0.04 g Trolox 100 g^−1^ DM were found for TP and TAA, respectively. The TP content and TAA in stems were already evaluated by other authors [23,26], but the differences in units of measurement they adopted hamper deeper comparisons. To the best of our knowledge, we studied for the first time changes in TP and TAA values caused by cooking in golden thistle midribs; in particular, we found a significant increase (*p* ≤0.01) in TP content for steaming (+19%) and ‘sous vide’ (+19%), as well as in TAA after ‘sous vide’ (+29%) treatments (Figure 2). Therefore, among the cooking methods considered in the present study, these results indicated ‘sous vide’ as the treatment able to better preserve the antioxidant properties of the cooked product. As previously reported for several vegetables [14,38,39,40,41], we found a good positive correlation (r^2^ = 0.74) between phenolic content and antioxidant activity. The scientific literature offers a very complex scenario about changes in TP content and TAA after different cooking processes. In general, the variability of data observed in the different species subject to cooking can be attributed both to different cooking parameters and to different vegetable matrices [42]. It is common to find papers in which the same cooking method causes different effects depending on the treated vegetable. For example, in a similar study on other edible herbs, Sergio et al. [14] reported that, after steaming, TP content increased in *Helminthotheca echioides* (+46%) and *Sonchus oleraceus* (+44%), remained stable in *Taraxacum officinale*, and decreased in *Urtica dioica* (−40%). In correspondence, they found a significant increase in TAA values in the first three species while a strong decrease in the last one, with a significant correlation (*p* < 0.001) between TP and TAA. Furthermore, an increase in TP content and stable TAA values were reported in two wild asparagus genotypes after steaming [43]. Finally, Renna et al. [33] did not find significant changes in TP content and TAA values in stems of two cultivars of chicory (*Cichorium intybus* L.; Catalogna group) after steaming and ‘sous vide’ cooking. The variable effect of similar cooking methods for treating different vegetables can be the result of the different structures of the plant tissue in the single species/genotypes, usually characterized by the variable extent of fibrosity and lignification [14]. Generally, boiling can easily lead to the loss of the phenolic component by leaching, although this loss can be partially compensated by the release of phenolic compounds from the walls of the plant tissue, which are progressively broken down by heat during cooking. The collapse of the cell wall structure of heated vegetable tissue could explain the increase in phenolic content and consequently also the antioxidant activity, in the case of steamed, microwaved, and ‘sous vide’ treated herbs, in which products are not soaked as happens for boiling [14,42]. Moreover, since each vegetable presents a different phenolic composition, it might be very useful to assess the effect of cooking on the individual phenolic compounds present in the vegetable.

### 2.4. Phenolic Composition and Content

**Figure 3:**
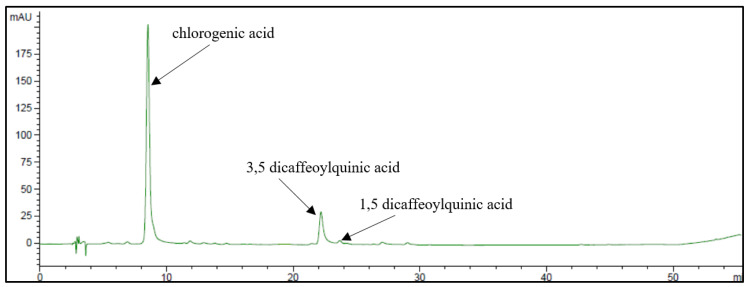
HPLC-DAD chromatogram of phenolic extract obtained from raw golden thistle midribs recorded at 325 nm.

**Figure 4:**
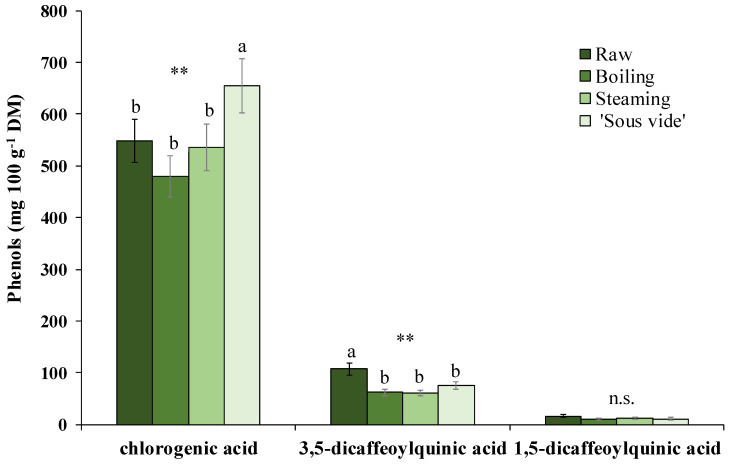
Main phenolic compounds identified in golden thistle midribs raw and cooked by boiling, steaming, and ‘sous vide’. Vertical bars indicate the standard deviation. Different letters indicate statistically different values (α = 0.05) between treatments. Significance: n.s., not significant; **, significant at *p* ≤ 0.01.

The phenolic pattern of golden thistle midribs was assessed by HPLC-DAD as described in Section 3.7. A typical chromatogram of a phenolic extract of golden thistle midribs is shown in Figure 3. Chlorogenic acid (CHLA) was the most abundant phenolic compound, followed by lower amounts of 3,5- and 1,5-dicaffeoylquinic acids (3,5- and 1,5-DCQA) (Figure 4); as flavonoids, traces of unidentified quercetin derivative and other unidentified compounds were found.

It is not easy to compare our data with information available in the literature on this subject due to both the different parts of the plant analyzed and the different extraction methods used. Nevertheless, our results are in good agreement with data reported by Marmouzi et al. [23], which performed a study on different parts of the plant (roots, stems, leaves, and flowers). In stems, these authors reported the presence of a good amount of CHLA, caffeic acid (probably coming from the hydrolysis of dicaffeoylquinic acids) and rutin (quercetin-3-rutinoside). In other papers, the phenolic composition was assessed on leaves or the whole aerial part. For example, in the aerial part, Sanz et al. [44] first identified a flavonoid derived from quercetin (quercetin-3-O-(2”-O-caffeoyl)- β-D-glucuronopyranoside), together with chorogenic, isochlorogenic, coumaric and protocatechuic acids and other quercetin and kaempferol derivatives. On the contrary, Petropoulos et al. [22] reported only the presence of flavonoids (derivatives of quercetin, luteolin, kaempferol, apigenin, and isorhamnetin) in the aerial part of the plant. Finally, in golden thistle leaves, Gonçalves et al. [45] found CHLA and kaempferol -3-glucoside as the main phenolics, together with a good amount of 4-O-cafffeoylquinic acid, 3,5-dicaffeoylquinic acid, quercetin-3-rutinoside and small amounts of other flavonoids (derivatives of quercetin, kaempferol, apigenin, and isorhamnetin).

Furthermore, as the effects produced by the different cooking methods on the content of individual phenolic compounds, a significant decrease was recorded in 3,5-DCQA content (−38% on average over the three treatments). On the contrary, a significant increase (+20%) in CHLA content (together with a +29% increase in TAA) was found in samples that underwent the ‘sous vide’ cooking treatment. Boiling and steaming treatments did not show significant changes for this compound (Figure 4).

Generally, the effects of different cooking methods on the content of individual phenolic compounds can be affected by two opposite causes: (i) thermal degradation and water leaching and (ii) matrix softening, which increases the extractability of phenols, resulting in a higher concentration than raw vegetables. Consequently, the structure of the plant tissue plays an important role in regulating these factors, so the final result can be significantly different not only in relation to the different cooking methods but also to the considered species and cultivar. Due to this complex scenario, conflicting results have been found in the literature [42].

As introduced above, no information is available in the literature regarding changes in the content of phenolic compounds of cooked golden thistle midribs. However, in a similar study on edible herbs, Sergio et al. [14] reported that CHLA content increased to a different extent after steaming as well as microwave (MW)-cooking in *Asparagus acutifolius*, *H. echioides*, *S. oleraceus*, *T. officinale*, and *Urospermum picroides*, providing a significant positive correlation (*p* < 0.01) with TAA values in the case of MW-cooking. In the same study, these authors found a drastic decrease in CHLA content and, as a consequence, of TAA in *U. dioica* after boiling, steaming, and MW-cooking. Moreover, a complete loss of CHLA was reported in boiled carrots by Palermo et al. [42] whereas, for fresh broccoli, Pellegrini et al. [46] reported a remarkable increase (about sevenfold) in CHLA content after oven steaming and a significant decrease (about −75%) after MW-cooking. According to this latter result, in fresh broccoli, Vallejo et al. [47] reported that both high-pressure boiling and MW-cooking caused considerable losses of caffeoylquinic acid derivatives by −47% and −87%, respectively.

Therefore, the results of our trials indicated CHLA as one of the bioactive compounds-certainly, not the only one-contributing to the antioxidant potential of golden thistle. Furthermore, as already reported for other species [14], in the present study, milder treatments than boiling, such as steaming and ‘sous vide’, reduced the loss or produced an increase in phenolic content and antioxidant capacity, then enhancing the nutraceutical value of processed products.

### 2.5. Sugars and Inulin

**Figure 5:**
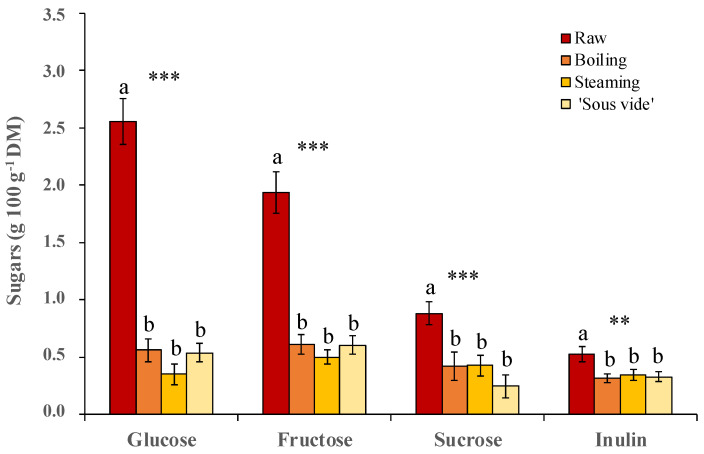
Content of glucose, fructose, sucrose, and inulin in golden thistle midribs raw and cooked by boiling, steaming, and ‘sous vide’. Vertical bars indicate the standard deviation. Different letters indicate statistically different mean values (*n* = 3) at α = 0.05 between treatments. Significance: ** and ***, significant at *p* ≤ 0.01 and *p* ≤ 0.001, respectively.

The sugar and inulin content as well as their changes depending on the different cooking methods, are shown in Figure 5. In the raw product, glucose is the most abundant sugar (2.6 ± 0.2 g 100 g^−1^ DM), followed by fructose (1.9 ± 0.2 g 100 g^−1^ DM) and sucrose (0.9 ± 0.1 g 100 g^−1^ DM), whereas the inulin content was 0.5 ± 0.1 g 100 g^−1^ DM. There is little evidence in the literature regarding the golden thistle sugar content. Recently, under different cultivation conditions, the presence of the abovementioned three simple sugars in the same order of abundance was reported [27], altought at about double amount we found. These authors also reported the presence of trehalose, which was not detected in our study. On the contrary, in our trials, we found a minimal content of complex saccharides containing fructose (fructans), which we have referred to and quantified as inulin. This last result is particularly important from a nutraceutical point of view, given the well-known health-beneficial prebiotic properties of fructans [48,49].

As expected, all the used cooking methods here evaluated caused a strong significant decrease (*p* ≤ 0.001) in the content of all simple sugars (−81%, −68% and −56% on average for glucose, fructose, and sucrose, respectively), as well as in the inulin content (−40% on average over the three treatments, *p* ≤ 0.01). None of the used cooking methods showed significant differences in terms of greater preservation of the content of each sugar in the final cooked product (Figure 5). This result, often observed also for other cooked vegetables, is principally due to the loss of these components by leaching, given their high water solubility [42,50].

### 2.6. Sensory Evaluation

The sensory profile of the raw product was not carried out as it cannot be consumed raw. On a hedonic scale from 1 (highly unacceptable) to 9 (highly acceptable), all samples achieved an acceptable score (close or above 6—slightly acceptable) for each parameter. Texture and odor were not statistically affected by cooking treatments, with average values of 6.2 and 6.8, respectively. Instead, the color value decreased passing from boiling to ‘sous-vide’ to steaming, with values from 8.1 to 5.7 (Table 2). The boiled golden thistle samples were judged the best product also for taste and overall acceptability compared to the products from the other two treatments (Table 2). Unlike other studies of cooking treatments applied to vegetables, in the case of golden thistle midribs, boiling met consumer acceptability better than the other processing treatments, especially for color, taste, and overall acceptability. In stem chicory, ‘sous vide’ has been shown to give the best results among cooked products for each parameter [33]. Even in asparagus, ‘sous vide’ microwaving proved to be the most satisfactory treatment [34]. In kalian-hybrid broccoli, the vacuum-based methods, especially ‘sous vide’ microwaving, showed the best sensory scores through a positive combination of broccoli stem softening with juiciness and slight crispness [51]. The specific result of this experiment could be attributed to the peculiarity of the studied vegetable, mainly consisting of central leaf veins (midribs) characterized by a high fibrous texture.

### 2.7. Microbiological Analysis

In this study, three cooking methods were compared to produce a golden thistle midrib ready-to-eat product. Since vegetables can be a source of food-pathogenic bacteria, the first safety attempts were related to defining the shortest useful treatment time to ensure the lowest microbiological risk for consumers. The challenge test was carried out only for the ‘sous vide’ method because all pathogenic cells contaminating midribs that are closed in the envelope together with the food product cannot be lost in the pot, as surely happens for boiling and steaming methods.

Since ‘sous vide’ cooking is largely considered to be a pasteurization process [52], any new attempt to employ this mild treatment for different foods needs the definition of several cooking parameters that must be experimentally validated. Even though many strains could be used as target microorganisms, we focused our attention on *Escherichia coli*, which is long time considered one of the main microbiological problems for ready-to-eat vegetables [53,54]. Since more than 95% of *E. coli* strains have a β-d-glucuronidase-positive reaction [55], the target strain here employed was the enterotoxigenic *E. coli* O18:H11 ATCC 35401, which grows as a blue colony onto TBX agar plates compared to *E. coli* O157:H7 strains that do not express β-d-glucuronidase activity [56] remaining white on TBX such as many other non-*E. coli* bacteria. Golden thistle midribs were dipped into a microbial cell suspension producing a sample at 5.45 ± 0.15 log cfu mL^−1^. This concentration was sufficiently higher than natural *E. coli* contamination of midribs measured in 1.75 ± 0.21 log cfu g^−1^ FM. After 6, 9, and 12 min of ‘sous vide’ cooking, no blue colonies were found on TBX agar plates, informing us that the concentration of viable cells was lower than 1 log cfu g^−1^ FM, achieving more than four magnitude order of reduction. However, when *E. coli* cell viability was measured by the MPN method, only some tubes of 6 min treated samples showed a visible microbial growth that was confirmed to be *E. coli* on TBX agar plates. The microbial survival was calculated in 0.36 ± 0.44 MPN mL^−1^, in contrast to not inoculated and untreated samples showing 110 ± 0.32 MPN mL^−1^. Since no LTB tube inoculated with midrib suspension from 9 min ‘sous vide’ treatment was positive for *E. coli*, as demonstrated by TBX agar plates, the cooking time of following experiments was fixed at 9 min. Renna et al. [15] defined the sous-vide microwave (SV-MW) parameters to inactivate *E. coli* in chicory stems. SV-MW cooking achieved a 6 log reduction of *E. coli* population after 90 s, but, at higher contamination level, less than 10 cfu g^−1^ of surviving cells were still found. Under experimental conditions employed in this study, the ‘sous vide’ cooking produced similar results only for 9 min treated golden thistle midribs cooked showing a lower thermal death rate when *E. coli* cells are dipped in boiling water compared to that found for microwave treated contaminated vegetables.

Once the treatment time was defined, it was possible to assess that natural microbial populations were largely inactivated by each of the cooking methods evaluated, as shown in Table 3. Mesophilic aerobic bacteria, yeasts and molds, and *Enterobacteriaceae* were not detected in all cooked samples.

**Figure 6:**
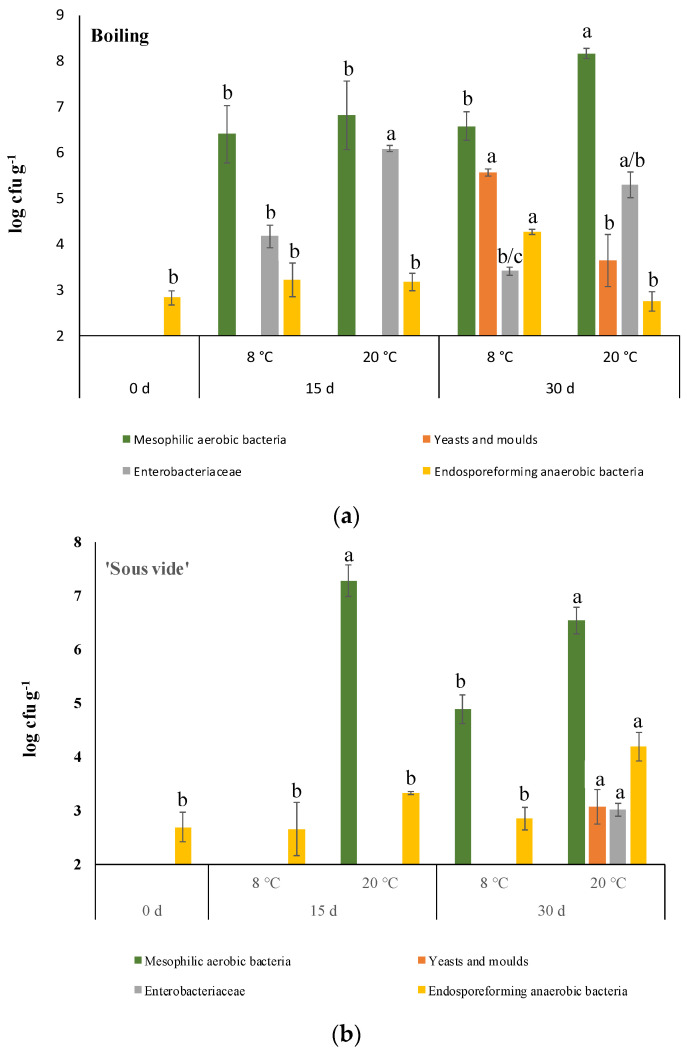
Comparison of viable cell densities of different microbial populations during 30 days of storage at 8 and 20 °C. Panel (**a**): boiled samples; panel (**b**): ‘sous vide’ samples. Limit of detection 2 log cfu g^−1^ FM. The lowercase letters for each microbial population during cold storage indicate statistical differences (*p* < 0.05) after a one-way analysis of variance (ANOVA) followed by the post hoc Duncan test.

Immediately after cooking, the sensory analysis of samples from steaming produced the lowest sensory score (Table 2), and for this reason, they were not considered for the evaluation of the shelf life. Boiled and ‘sous vide’ treated samples were then stored at 8 and 20 °C in order to define the combination cooking method/temperature of storage able to better preserve the microbial quality of this new kind of ready-to-eat product. As shown in Figure 6, the effect of storage at 20 °C sharply promoted microbial growth. In fact, in both boiled and ‘sous vide’ treated samples, mesophilic aerobic bacteria populations resulted in higher than 6 log cfu g^−1^ FM already after 15 days. The extension of the shelf-life period at this temperature resulted in a worsening of microbial quality for both cooked samples leading to the conclusion that this temperature is inappropriate for storing this ready-to-eat product. As expected, storage at 8 °C delayed microbial growth for the four populations considered in this study, allowing better differentiation between the two cooking methods. At refrigeration temperature, *Enterobacteriaceae* and mesophilic aerobic bacteria were detected already after 15 days of storage. After the same storage period, both microbial populations were not detectable (limit of detection = 2 log cfu g^−1^ FM) in ‘sous vide’ refrigerated samples. The extension of cold storage up to 30 days resulted in the growth of all microbial populations considered in this study only in boiled samples. On the contrary, only mesophilic aerobic bacteria were grown in ‘sous vide’ treated samples on day 30. As is normal for wild vegetables, endospore-forming anaerobic bacteria were always detected. However, they remained almost stable during shelf-life, reaching about 4 log cfu g^−1^ FM only after 30 days of storage.

Different studies evaluated the effect of several cooking methods on the microbial quality of ready-to-eat vegetables. Rinaldi et al. [57] evaluated the effect of ‘sous-vide’ cooking and steaming on the microbiological quality of carrots and broccoli during cold storage. They found a similar effect of the two cooking methods against total aerobic bacteria, yeasts and molds, and spore formers bacteria in the case of carrots and a better preserving effect of the sous-vide cooking in the case of broccoli. Sergio et al. [58] found that the cooking method (blanching or microwave) did not affect the load of mesophilic bacteria, yeasts and molds, and *Enterobacteriaceae* of semi-dried asparagus spears during cold storage. In green beans, microwave cooking followed by storage at 2 °C delayed the growth of aerobic mesophilic bacteria in comparison to the hot-water treatment [59].

Among the microbial population investigated, only the endospore-forming anaerobic bacteria survived all cooking methods. This result was expected since endospore-forming bacteria display a wide range of heat resistance and, having the soil as the main reservoir [60], they are normal contaminants of fruits and vegetables such as zucchini [61], different kind of vegetable salads [62] and spices [63]. Due to the heat resistance of endospore-forming bacteria, their grown under refrigerated conditions in ‘sous vide’ processed foods were considered for several foods such as mushrooms and shellfish salad [64], carrots and Brussels sprouts [57], chicken breast [65], and salmon slices [66]. Similarly to these studies, in ‘sous vide’ cooked golden thistle midribs, the endospore-forming bacterial population surviving the heat treatment remained almost stable at low temperatures. Microbiological evaluations carried out following two cooking methods and two temperatures for storage allowed us to define the best result for ‘sous vide’ processed golden thistle midribs stored in the bag under vacuum at 8 °C.

### 2.8. PCA

**Figure 7:**
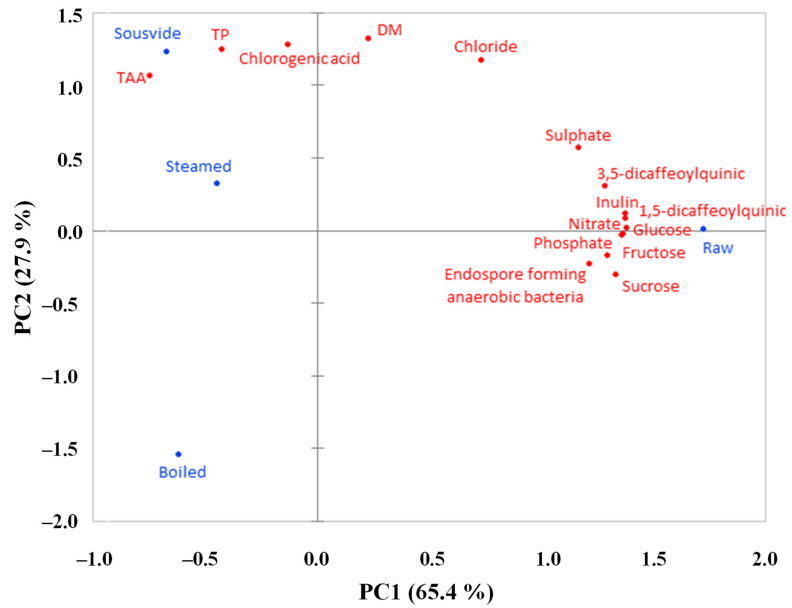
Analysis (PCA) describing the spatial distribution of DM, microbiological and chemical parameters for golden thistle midribs raw and cooked by boiling, steaming, and ‘sous vide’. DM = dry matter; TP = total phenols; TAA = total antioxidant activity.

In the PCA, the first two principal components (PC) explained 93.3% of the total variance, split as 65.4% and 27.9% between PC1 and PC2, respectively (Figure 7). A clear separation of variables between raw and cooked products was shown along the PC1, the raw samples being associated with all phenolic compounds except CHLA, all sugars and inorganic anions, and the endospore-forming anaerobic bacteria, which were all together higher in the raw product and generally decreased by the cooking process. In this sense, PCA is typically a visual representation of the ANOVA results as it takes all the data previously discussed through a comprehensive graph. Pearson’s correlation matrix shows that all of these parameters are highly and significantly correlated with each other (r > 0.95). All cooking methods were negatively correlated with PC1 and associated with TP, TAA, and CHLA, which were in turn positively correlated to PC2, together with steaming and ‘sous vide’ (Figure 7). Boiling instead is negatively correlated to PC2, perhaps due to the lower values found in boiled samples for TP, CHLA, DM, and Cl^-^ (Figure 2 and Figure 4; Table 1).

## 3. Materials and Methods

### 3.1. Chemical Reagents

Reagents. 2,2′-Azino-bis(3-ethylbenzothiazoline-6-sulfonic) acid (ABTS), ethanol (absolute, 99.8%), 6-hydroxy-2,5,7,8-tetramethylchromane-2-carboxylic acid (Trolox; 97%), methanol, potassium persulfate (99.0%), sodium bicarbonate (99.7%), sodium carbonate (anhydrous, 99.95%), and sodium nitrate (99.0%) were purchased from Sigma-Aldrich (Milan, Italy). Deionized water (resistivity = 18.2 MΩcm^−1^) was produced in a laboratory using a Milli-Q system (Millipore, Bedford, MA, USA). All reagents were of analytical grade, except methanol (HPLC grade).

HPLC standards. Caffeic acid (CA), chlorogenic acid (5-O-caffeoylquinic acid, CHLA), 1,5- and 3,5-dicaffeoylquinic acid (1,5- and 3,5-DCQA) were purchased from PhytoLab GmbH & Co. KG (Vestenbergsgreuth, Germany). Glucose, fructose, sucrose, potassium chloride, potassium nitrate, potassium dihydrogen phosphate, and potassium sulfate were purchased from Sigma-Aldrich (Milan, Italy). All HPLC standards had ≥95% chromatographic purity.

### 3.2. Sample Preparation and Cooking

Golden thistle plants were collected at the “Ditaranto” farm localized close to Gravina in Puglia (BA), Italy (40°50′40′’ N–16°21′36′’ E). The plant material was obtained from cultivation trials where was applied nitrogenous fertilization (100 kg ha^−1^ N) with ammonium nitrate split twice (40% at transplanting and 60% after 40 days) and a supporting irrigation regime. The seedlings used for the transplant came from autochthonous wild thistle seeds collected in the neighboring areas of the same farm. Golden thistle was planted in rows 0.5 m apart with a density of 6 plants m^−2^. Immediately after harvesting, which occurred in December, the root and basal leaves of the plant were removed with a sharp knife; then the leaf blade was manually removed because it is not edible for the presence of thorns; the remaining part of the plant represents the edible portion (Figure 1B). After these preliminary treatments in the field, the edible material was transferred to the laboratory under refrigerated conditions (8.0 ± 1.0 °C), where it was washed with tap water and dried by gently dabbing it with paper towels. Two batches were selected, the first one for the analysis of the raw samples (1 kg) and the second one for the analysis of the cooked samples (6 kg), including the sensory analysis. Each sample of about 200 g was prepared by dividing the single plant into at least 3 parts to randomize the sample and preserving the external appearance of the product for its use in particular culinary preparations; moreover, three replicates for each cooking treatment were prepared in order to obtain independent replications for each treatment, both raw and cooked.

**Figure 8:**
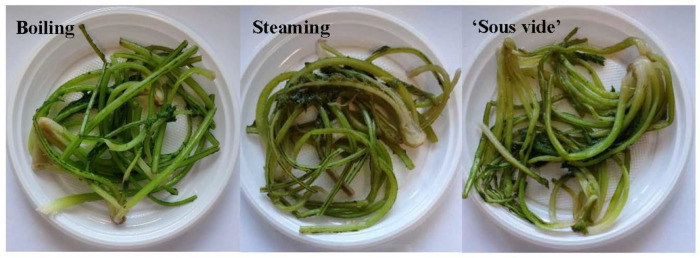
Golden thistle midribs cooked with different methods: boiling, steaming, and ‘sous vide’.

The plant material was then subjected to different cooking methods (boiling, steaming, and ‘sous vide’) (Figure 8). Optimal cooking conditions were established according to preliminary trials. In particular, the following cooking parameters were adopted:

Boiling: each sample (200 g) was immersed in 2 L of boiling tap water (99.0 ± 1.0 °C) contained in a covered stainless steel pot covered with a lid and cooked on an electric heating plate (ARED Heating Magnetic Stirrer, Velp Scientifica, Usmate Velate, MB, Italy). Cooking time: 8 min.

Steaming: each sample (200 g) was placed on a tray in a steamer cooker (VC 101 630 Tefal, Italy), equipped with a tank containing 1 L of tap water, covered with a lid, and cooked with water vapor (99.0 ± 1.0 °C) under atmospheric pressure. Cooking time: 17 min.

‘Sous vide’: each sample (200 g) was sealed in a vacuum chamber (Ecovac, Fiorenzuola d’Arda, Piacenza, Italy), using 25 cm × 35 cm vacuum packing bags (Wimex S.r.l., Laives, Bozen, Italy), with upper side smooth and a lower side embossed (outside: nylon; inside: polyethylene), and cooked as described in boiling treatment. Cooking time: 9 min.

### 3.3. Dry Matter of Raw and Cooked Golden Thistle

Fifty grams for three replicates were weighed individually before cooking. After cooking, samples were dried in a forced draft oven at 65 °C until constant weight. Results were expressed as a percentage of dry matter (DM) referred to as the raw sample weight before cooking.

### 3.4. Inorganic Anion Content

The analysis was performed using an ion exchange chromotagraph (Dionex DX120; Dionex Corporation, Sunnyvale, CA, USA) with a conductivity detector, as reported by D’Imperio et al. [67]. Inorganic anionic content (Cl^−^, NO_3_^−^, H_2_PO_4_^−^, SO_4_^2−^) was determined in 0.5 g dried sample ground to powder using an IonPac AG14 precolumn and an IonPac AS14 separation column (Dionex Corporation). All analyses were performed in triplicate.

### 3.5. Preparation of Phenolic Extracts

Three sub-samples of 25 g each were taken from the raw and cooked samples; after homogenization, they were extracted (twice for 1 h) under reflux in a hot water bath with boiling methanol (1:10 *w/v*). The combined methanolic extracts were concentrated under reduced pressure by a rotary evaporator. The obtained residue was dissolved in 50% (*v/v*) methanol/water; the resulting solution was filtered through a Whatman Grade 1 filter paper and used for: (i) the evaluation of total phenolic concentration; (ii) the HPLC analysis of phenolic compounds; and (iii) the antioxidant activity assay. All details of the extraction procedure were as already reported by Sergio et al. [14].

### 3.6. Total Phenolic Content

Total phenolic (TP) content was determined on phenolic extracts using the Folin-Ciocalteu method, as reported by Gatto et al. [68]. Caffeic acid (CA) was used as a reference standard for the calibration curve; phenolic content was estimated as CA equivalent (CAE) and expressed as mg CAE g^−1^ DM. All analyses were performed in triplicate.

### 3.7. HPLC-DAD Analysis of Phenolic Compounds

The analysis of phenolic compounds was performed on the same extracts used for TPC evaluation. Individual phenolics were evaluated by HPLC using an Agilent 1100 Series liquid chromatograph (Agilent Technologies Inc., Santa Clara, CA, USA) equipped with a binary gradient pump (Agilent P/N G1312A) and spectrophotometric photodiode array detector (DAD) (Agilent P/N G1328A); data were processed using the Agilent ChemStation (Rev. A.06.03) software. All chromatographic details were reported by Gatto et al. [69]. All analyses were performed in triplicate.

### 3.8. Antioxidant Capacity Assay

Total antioxidant activity (TAA) was assayed by the radical cation ABTS assay on the same extracts used for TPC evaluation, as described in detail by Sergio et al. [43]. The antiradical activity was expressed as g Trolox 100 g^−1^ DM. All analyses were performed in triplicate.

### 3.9. Sugar and Inulin Content

Three sub-samples of 25 g each were taken from the raw and cooked samples; after homogenization, they were extracted (twice for 1 h) under reflux in a hot water bath with boiling deionized water (1:10 *w*/*v*). The extract was filtered through a Whatman Grade 1 filter paper and analyzed for glucose, fructose, and sucrose content using a Dionex chromatographic system (ED40 electrochemical detector, GP50 gradient pump, PeakNet 5.11 software) and a CarboPacPA1 column (4 mm × 250 mm) (Dionex Corporation, Sunnyvale, CA, USA). The chromatographic conditions and further experimental details were as already reported [40]. Glucose, fructose, and sucrose content were expressed as g 100 g^−1^ DM. All analyses were performed in triplicate.

The inulin content was determined on the same extract used for sugar analysis, as described by Sergio et al. [50]. Briefly, an aliquot of this solution was hydrolyzed with hydrochloric acid; the resulting solution was analyzed for glucose and fructose content by HPLC, as described above. The inulin content was calculated from the content of simple sugars before and after hydrolysis and expressed as g of fructose 100 g^−1^ DM [70]. All analyses were performed in triplicate.

### 3.10. Sensory Evaluation

The plant material subjected to the three cooking methods was used for the sensory analysis, involving a selected group of seventeen assessors (made up of 7 females and 10 males, aged between 25 and 64 years old), previously trained in the descriptive analysis of vegetables. Color, taste, odor, texture, and overall acceptability of samples were assessed using a 9-point hedonic scale, with 9 as the best value [71]. During the analysis, the material (about 30–50 g) was presented to the panelists at room temperature under normal lighting conditions in plastic dishes, coded using random, three-digit numbers. The scores of the sensory evaluations were used in the statistical analysis.

### 3.11. Microbiological Analysis

Vegetable samples (25 g) were shredded with sterile scissors, homogenized with sterile saline solution, and decimally diluted to enumerate total mesophilic aerobic bacteria, *Enterobacteriaceae,* and yeasts and molds as previously reported [72,73]. In order to enumerate anaerobic endospore-formers, 10 mL of vegetable suspension was pasteurized at 80 °C per 10 min and immediately dipped in melting ice. Aliquots of 100 µL of decimal dilutions in peptone-cysteine buffer at pH 7,2 were promptly spread onto pre-reduced Differential Reinforced Clostridial Medium (DRCM) plates that were incubated up to 4 days at 30 °C in the anaerobic cabinet (Le Petite Glove box, Plas-Labs, Inc, Lansing, MI, USA) filled with a 90% N_2_ + 10% H_2_ atmosphere. The same microbial populations were enumerated in packed boiled and ‘sous vide’ samples stored at 8 and 20 °C during 30 days of storage. Results were expressed in log cfu g^−1^ FM. All microbiological media, as well as cycloheximide and chloramphenicol selective solutions, were purchased from Biolife Italiana srl (Milan, Italy). The safety of ‘sous vide’ processed golden thistle samples was presumptively defined, establishing the lower time exposure necessary to kill at least four log cfu mL^−1^ of the enterotoxigenic *Escherichia coli* O18:H11 ATCC 35,401 strain. Twenty-five mL of sterile saline solution was inoculated with 1% of standardized cell suspension [74], in which 50 g of golden thistle shredded midribs were dipped. Contaminated and not inoculated samples (vegetable tissue and saline solution) were sealed and processed by ‘sous vide’ for 6, 9, and 12 min. Inoculated and untreated samples were stored. After the treatment, samples were placed on ice and supplemented with 75 mL of cold, sterile saline solution. Samples were then homogenized as described above and analyzed according to ISO 16649-2:2018 [75], expressing *E. coli* load in log cfu g^−1^ FM after 48 h at 44 °C. Lauryl Tryptose Broth (LTB, Biolife Italiana) was inoculated, in triplicate, with 1 mL of homogenized vegetable containing 0.1, 0.01 and 0.001 g of vegetable tissue in order to estimate *E. coli* load by most probable number (MPN) method as required by the ISO 7251:2005 [76]. The presumptive *E. coli* cells grown in LTB were verified onto TBX agar plates.

### 3.12. Statistical Analysis

The experiment was a one-factor (treatment) design. All data were subjected to ANOVA (GLM procedure, SAS software, SAS Institute, Cary, NC, USA), separating means by Student-Newman-Keuls (SNK) test. For visual analysis of data, Principal Component Analysis (PCA) (PRINCOMP procedure, SAS software) was performed on mean-centered and standardized (unit variance scaled) data prior to analysis. The data matrix processed in PCA was composed of four observations (three cooking methods plus raw products) and 15 variables. Only the data allowed to obtain a complete data matrix have been considered in PCA, thus excluding three of the microbiological variables with missing values in the cooked products and the sensory analysis data, which do not include the raw product.

## 4. Conclusions

Wild edible plants are gaining more and more interest as alternative/complementary food sources, a trend supported by increasing scientific studies for their valuable health effects and functional properties. Taking into account that a large part of young people is more concerned and aware of health issues and is willing to adopt a healthy lifestyle, this study tries to reduce the knowledge gap between generations by presenting traditional food as a ready-to-eat product. The results of this study also contribute to reducing the loss of traditional knowledge on uses and recipes concerning wild herbs and to encourage further studies on species not yet sufficiently investigated as their potential in food applications.

## Figures and Tables

**Table 1:** Dry matter, weight change, and inorganic anion content in raw and cooked golden thistle midribs.

Treatments	DM	Weight Change	NO_3_^−^	SO_4_^−2^	Cl^−^	H_2_PO_4_^−^
	(g 100 g^−1^ FM) ^#^	%	(mg g^−1^ DM)	(mg g^−1^ DM)	(mg g^−1^ DM)	(mg g^−1^ DM)
Raw	5.7 ± 0.1 a		69.1 ± 2.3 a	1.2 ± 0.24	60.2 ± 1.6 a	15.4 ± 1.1 a
Boiling	4.7 ± 0.1 b	−5.0 ± 1.2 a	38.4 ± 1.3 b	0.9 ± 0.07	35.2 ± 0.6 b	8.3 ± 0.2 c
Steaming	5.9 ± 0.1 a	−9.9 ± 0.9 b	41.5 ± 0.8 b	1.1 ± 0.04	51.6 ± 5.1 a	10.4 ± 0.5 b
‘Sous vide’	6.0 ± 0.3 a	−8.4 ± 1.4 b	37.8 ± 1.6 b	1.0 ± 0.12	57.9 ± 6.5 a	6.3 ± 0.4 d
*Significance ^(1)^*	***	**	***	ns	***	***

^(1)^ ns, not significant, **, *** significant at *p* ≤ 0.01 and *p* ≤ 0.001, respectively. Different letters indicate mean values (*n* = 3) statistically different at α = 0.05. DM = dry matter. FM = fresh matter. **^#^** %DM referred to the raw sample weight before cooking.

**Table 2:** Sensory analysis of golden thistle midribs cooked with different methods. Evaluation of samples resulted from scoring five macro-descriptors with the hedonic scale having a scale from 1 (highly unacceptable) to 9 (highly acceptable). Mean values ± SD.

Treatment	Color	Odor	Taste	Texture	Overall Acceptability
Boiling	8.1 ± 0.8 a	7.0 ± 1.1	6.9 ± 1.3 a	6.6 ± 1.5	7.5 ± 1.1 a
Steaming	5.7 ± 1.1 c	6.4 ± 1.5	5.9 ± 1.2 b	6.2 ± 1.2	5.9 ± 1.3 b
‘Sous vide’	6.8 ± 1.0 b	7.1 ± 1.1	6.2 ± 1.3 ab	5.8 ± 1.3	6.3 ± 1.3 b
*Significance ^(1)^*	***	ns	*	ns	***

^(1)^ ns, not significant, *, *** significant at *p* ≤ 0.1 and *p* ≤ 0.001, respectively. Different letters indicate mean values (*n* = 3) statistically different at α = 0.05.

**Table 3:** Viable cell densities of different microbial populations in raw and cooked golden thistle midribs.

Treatment	Mesophilic Aerobic Bacteria	Yeasts and Moulds	*Enterobacteriaceae*	Endospore-Forming Anaerobic Bacteria
	(log cfu g^−1^ FM)	(log cfu g^−1^ FM)	(log cfu g^−1^ FM)	(log cfu g^−1^ FM)
Raw	6.91 ± 0.11 a	4.83 ± 0.21 a	6.23 ± 0.08 a	3.63 ± 0.07 a
Boiling	ND b	ND b	ND b	2.83 ± 0.16 b
Steaming	ND b	ND b	ND b	2.30 ± 0.36 b
‘Sous vide’	ND b	ND b	ND b	2.69 ± 0.27 b
*Significance ^(1)^*	***	***	***	***

^(1)^ *** significant at *p* ≤ 0.001. Different letters indicate mean values (*n* = 3) statistically different at α = 0.05. FM = fresh matter. ND: not detected. Limit of detection 2 log cfu g^−1^ FM.

## Data Availability

Data is contained within the article.
